# O1-4 Validation of questions to assess the mode of commuting to/from school for children according to their parents: the PACO study

**DOI:** 10.1093/eurpub/ckac094.004

**Published:** 2022-08-29

**Authors:** María Jesús Aranda Balboa, Marina Fernández, Emilio Villa-González, Berta Murillo-Pardo, José Manuel Segura-Diaz, Romina Gisele Saucedo-Araujo, Yahira Barranco-Ruiz, Manuel Herrador-Colmenero, Francisco Javier Huertas-Delgado, Palma Chillón

**Affiliations:** 1 Department of Physical Education and Sport, Faculty of Sport Sciences, University of Granada, Granada, Spain; 2 Department of Physical Education and Sport, Faculty of Education and Sport Sciences, University of Granada, Melilla, Spain; 3 Department of Education, Faculty of Education, University of Zaragoza, Zaragoza, Spain; 4 Department of Physical Education and Sport, Teacher Training Centre La Inmaculada University of Granada., Granada, Spain

**Keywords:** Children, adolescents, adults, surveys, active transportation

## Abstract

**Background:**

The active commuting to school behaviour is an opportunity to increase the physical activity levels. The most frequent used tool to assess the mode of commuting to school is self-reporting by children or by their parents. However, there is a lack of information about the validity between both, children and parents. So, the purpose is to validate the questions of mode of commuting to go and come back from school of children according to their parents'.

**Method:**

A total of 611 parents (mean age: 43.28±6.25 years old) and their children (mean age: 11.44±2.77 years old) from Granada (Spain) completed a family (mode of commuting of children reported by parents as part of the Family PACO questionnaire) and a student questionnaire validated (Chillon et al., 2017) (mode and frequency of commuting to and from school questionnaire), respectively, in two measurement points. The questions from the ‘Family PACO questionnaire' are: “How does your child usually go to school?” and “How does your child usually come back?”; and the questions from the ‘mode and frequency of commuting to and from school questionnaire' are: “How do you usually go to school?” and “How do you usually go from school?”. The validation between parents and childreńs questions was analysed using Kappa and Spearman correlation coefficients. The results of the kappa are considered as: poor agreement (0-0.20), acceptable agreement (0.21-0.40), moderate agreement (0.41-0.60), substantial agreement / good (0.61-0.80) and almost perfect / very good agreement (0.81-1.00) (Landis & Koch, 1977). The Spearman correlations coefficients were interpreted as low (> 0.30), moderate (0.30-0.50), and high (> 0.50) (Van Dyck, Cardon, Deforche, & De Bourdeaudhuij, 2015).

**Results:**

The validity of questions from both questionnaires about mode of commuting presented high coefficients of validation (Kappa coefficient; 0.865 to school and 0.839 from school and Spearman correlation; rho=0.882 to school and rho=0.860 from school).

**Conclusion:**

The questions about the mode of commuting to/from school from the ‘Family PACO questionnaire’ are valid method. Therefore, the use of the questions would be recommended to assess children's mode of commuting.

